# Potential impacts of microplastic pollution on soil–water–plant dynamics

**DOI:** 10.1038/s41598-025-93668-0

**Published:** 2025-03-21

**Authors:** Alireza Bakhshaee, Peyman Babakhani, Muhammad Masood Ashiq, Kati Bell, Maryam Salehi, Farhad Jazaei

**Affiliations:** 1 Department of Civil, Construction & Environmental Engineering, University of Memphis, Memphis, TN USA; 2 Ground Engineering, University of Manchester, Manchester, UK; 3 Research and Innovation, Brown and Caldwell, Nashville, TN USA; 4 Department of Civil and Environmental Engineering, University of Missouri, Columbia, MO USA; 5 Center for Applied Earth Science and Engineering Research, University of Memphis, Memphis, TN USA

**Keywords:** Plastic contamination, Soil hydrology, Microplastic morphology, Soil hydraulic properties, Hydrogeology, Environmental impact, Hydrology

## Abstract

This study was designed to assess the potential impact of microplastic (MP) pollution on soil hydrology, specifically in retaining and releasing moisture. Herein, High-Density Polyethylene (HDPE) MP of different sizes (i.e., 0.5–1, 1–3, and 3–5 mm) and shapes (i.e., fiber, film, and fragment) were evaluated for their effects on water retention curve (WRC) of sandy loam soil, chosen for its agricultural relevance and widespread environmental presence of HDPE. Nine contamination scenarios were simulated with a low MP pollution rate, 0.01% *w/w*. Van Genuchten models were used to assess plant available water (PAW), wilting point (WP), and water holding capacity (WHC). Results showed that studied MP could significantly affect WRC and PAW mainly by changing WHC rather than WP and that this effect varied with MP shape and size. According to the results, fragment MP had the greatest impact on soil WHC by increasing 36.3%, followed by fibers and films by 19.8% and 15.7%. MP particles significantly increased WHC, while WP remained relatively unchanged. An observed trend indicated that the impact on WHC increased with the size of the MP particles. These findings emphasize the need to manage soil MP pollution to protect plant growth, agriculture, and water dynamics.

## Introduction

The use of plastics is widespread in society because of their desirable properties, such as malleability, durability, and cost-effectiveness^1–3^. It is estimated that the yearly rate of plastic production has increased exponentially, from 1.5 to 367 million tons from 1950 to 2021^4,5^. This rapid and excessive production of plastic, as well as the substantial amount of waste that is discharged into the environment, has raised important concerns^6–9^ related to the pollution of oceans and waterways, ecological damage to wildlife and soil fauna, and the possibility of plastic entering the food chain and posing health risks to humans^10–17^. Microplastic (MP) particles are usually defined as plastic pollutants with a size range of 1 μm to 5 mm, although there are slightly different interpretations of the term in some articles^18^. This includes both primary plastics, those made purposefully, and secondary plastics, those that occur as a result of the breakdown and degradation of larger plastic pieces^19–21^.

MP particles have been found in almost every marine and terrestrial environment, including mountains and Antarctica^22–24^. Nevertheless, several recent studies have reported that agricultural farms are among the most highly MP-contaminated areas in terrestrial systems^9,25,26^. Generally, farms can become contaminated by MP particles from various routes, including plastic mulching, biosolid fertilizers, and MP-polluted irrigation water^27–33^.

The impact of MP pollution has been explored by a variety of disciplines, including biology, soil chemistry, microbial ecology, plant sciences, and environmental toxicology, for example, soil health, nutrient cycling, and soil–plant-microbe interactions^34–46^. Some studies also focused on MP pollution impacts on the physical characteristics of soil, such as soil compressibility, stability, porosity, and bulk density^47–49^. Apart from these studies, relatively few have examined the impact of MP on the dynamics between soil, water, and plants, commonly referred to as soil hydrology. This involves how MP particles can change the mechanism by which soil releases water to plant roots and the atmosphere and stores it within its pore spaces^50–52^. It is crucial to emphasize that even minor changes in soil–water–plant dynamics, especially when scaled up across larger areas like watersheds and agricultural regions, can have strong domino effects on hydrological processes, including disturbances in watershed water balances (e.g., evapotranspiration, groundwater recharge, infiltration, and runoff rates, and required irrigation withdrawals), as well as biological impacts on plants, landcover, and ecosystems^34,38,53–56^.

Within the soil hydrology context, one study found that increasing polyethylene MP pollution concentrations (0.5–3% *w/w*, 0–500 μm) reduced saturated conductivity and evaporation rates in saline soil, while another reported that polyethylene plastic films (1% *w/w*, 2–10 mm) influenced water evaporation and desiccation cracking in clay soils^57,58^. Furthermore, research on the effects of MP shape (fiber, strand, pellet) and concentration (0.5–3% *w/w*, ~ 3 mm) in fine sand revealed that MP particles significantly affected water holding capacity and late-stage evaporation with impacts varying by shape and concentration^59,60^. Additionally, a study on mixed shape and size (0.5–3 mm) polyethylene terephthalate and polystyrene MP particles at higher concentrations (0.5–2% *w/w*) in loess topsoil from agricultural Luvisol found that increasing MP concentrations reduced both hydraulic conductivity and soil water retention, regardless of MP type^47^. Additionally, research on polyethylene MP particles at varying concentrations (0.5%, 1%, 2%) and sizes (150, 550, and 950 μm) in sandy and loamy soils showed that while low concentrations (0.5% *w/w*) had minimal effects, higher concentrations (2%) significantly altered water-holding properties. Smaller MP particles (150 μm) enhanced water retention in loamy soil and sandy soil^61^. Lastly, a study on polypropylene MP (20, 200, 500 μm; up to 6% *w/w*) in loam, clay, and sand soils found that MP particles reduced saturated hydraulic conductivity and water retention capacity in all soils, with the most significant reduction in water retention observed in clay^62^.

Soil–water–plant dynamics, recognized for their complexity, have been extensively studied for decades^63^. These dynamics are influenced by a range of factors, including soil type, natural organic matter (NOM) content, aggregate hydrophobicity, drying and wetting cycles, and micropore structural properties such as porosity, bulk density, pore connectivity, and desiccation cracks development^64–70^. Previous studies have indicated that MP morphology—including shape (e.g., fragment, film, fiber, foam), size (~ 10 μm to 5 mm), and surface hydrophobicity—affects soil hydrology by altering a range of factors, including soil pore structure, preferential flow paths, hydraulic connectivity of pores, and water molecule interactions with solid particles^57,59,61,62^. Therefore, minimizing variability in these factors is essential to isolate and accurately evaluate their individual impacts on soil hydrology under MP pollution, though addressing this complexity poses a persistent challenge.

Building on previous findings, the main objective of this study was to enhance our understanding of the impacts of MP pollution on soil–water–plant dynamics in terms of soil water retention curve (WRC), emphasizing critical parameters such as the plant-available water (PAW), water-holding capacity (WHC), and wilting point (WP), without delving into their biological or ecological consequences^53,71–74^. The study sought to address some limitations of previous research. First, it focused on a lower MP concentration (0.01% *w/w*), which is a more environmentally relevant rate compared to the higher concentrations (0.5–6% *w/w*) commonly studied, standing for excessively contaminated areas or potential future conditions. This approach enhances the generalizability of the findings. Second, it ensured precise control over the shape and size of MP particles, using similarly shaped particles only scaled in size to minimize uncertainty, unlike prior studies that often used random or unconsidered shapes. Third, in contrast to prior research, this study carefully accounted for the potential presence of background MP particles in the soil by implementing thorough cleaning procedures to eliminate any pre-existing MP contamination. Fourth, to minimize uncertainty, this study implemented triplicate analyses on a single sample instead of utilizing multiple samples. This methodology mitigates variability arising from the spatial heterogeneity of MP and NOM particle distribution within the soil matrix, a critical factor often neglected in prior research^70,75^. As detailed in the Method section, this study focused on sandy loam soil and examined the effects of high-density polyethylene (HDPE) at a concentration of 0.01% (*w/w*). Sandy loam was selected for its widespread use in agriculture and its relevance to real-world farming conditions. This soil type is highly versatile and supports the cultivation of a diverse range of crops, including vegetables (e.g., carrots, lettuce, tomatoes), fruits (e.g., strawberries, melons, citrus), cereals (e.g., wheat, maize), and legumes (e.g., beans, peas)^76^. HDPE was selected due to its widespread use globally and its significant presence in agricultural environments through sources like biosolids, stream water, and farm applications such as irrigation pipes, plastic mulch films, greenhouse covers, and packaging materials^77–79^.

## Methods

### Microplastics

MP particles found in the environment occur in a variety of shapes, such as fragments, fiber, film, foam, and pellets in different sizes^80,81^. Plastics can be manufactured from a variety of polymers, and high-density polyethylene (HDPE) is the most predominant plastic polymer due to its versatile properties and wide uses^82–84^. Therefore, in this study, we investigated the impacts of HDPE MP with different shapes (i.e., , fiber (Fig. SI-10), film (Fig. SI-11), and fragment (Fig. SI-12)) and sizes (i.e., 0.5–1 mm (small MP), 1–3 mm (medium MP), and 3–5 mm (large MP)), on soil WRC, WHC, PAW, and WP, as the major quantitative measures used in soil, agriculture, and hydrological sciences for studying soil hydrology^73,85–88^. The selected shapes and sizes represent common MP sources, such as torn and fragmented films (e.g., plastic mulch), synthetic textiles (e.g., ropes and agricultural mats), and fragmented plastic debris (e.g., irrigation pipes and plastic containers), frequently encountered in agricultural settings^84,89^. Figures SI-8 and SI-9 illustrate the careful cutting and production process of HDPE plastic rods to achieve the exact size range. Additionally, biosolids—organic fertilizers derived from treated sewage sludge—are known to contain MP particles originating from urban areas, including fibers from textiles and fragments and films from household plastics^27,31,90^. Moreover, this shape and size categorization aligns with widely used MP classifications in the literature, ensuring consistency and comparability across studies.

To control the variables under investigation (i.e., plastic type and chemistry), all MP particles were generated from a single HDPE rod purchased from Duco Plastics & Supply LLC, Pennsylvania, USA. The Supplementary Information (SI) section provides detailed technical information regarding the method employed to create the MP with specific shapes and sizes. Fig. SI-1a displays Fourier-transform infrared (FTIR) spectroscopy results, confirming them as HDPE and Fig. SI-1b shows three ranges of MP and shapes used in this study. As a measure of hydrophilicity, the water contact angle was assessed using the Ossila contact angle goniometer, resulting in the value of determined 67 ± 10° (Fig. SI-1c), which refers to very weak hydrophilicity^91,92^. According to previous studies, chemically treated HDPE and those with rough surfaces can exhibit weak hydrophobicity^93–95^. Soil hydrophilic compounds attract and retain water molecules, forming water films that facilitate water movement and storage within soil aggregates. Furthermore, this may affect water distribution and flow dynamics by introducing heterogeneity in soil wettability^96,97^.

In this study, we used natural agricultural soil collected from the Agriculture Center Memphis located at 35°7′29″ N– 89°48′23″ W. To minimize the presence of MP background in the soil and reduce the risk of pre-contamination by MP, we collected soil samples from a depth of over 30 cm in a pristine natural area far from farming plots where MP particles might have already contaminated the land^9,16^. In preparation for experiments, soil samples were analyzed, and quality assurance and control measures were employed, as described below.

#### Natural organic matter (NOM) content

Agricultural NOM from plant and animal residues enhances WHC, retaining moisture and reducing evaporation^98–101^. In this study, the NOM content of the field soil was assessed in the laboratory by combusting it in a furnace at 570 °C for 3 h; results showed the NOM content of 3% (*grams per gram*)^102^.

#### Soil baseline characteristics

Here, the advanced PARIO Particle Size Analyzer manufactured by METER, which determines soil particle size distribution (PSD) based on Stokes’ law, with particle sizes ranging from 63 to 1 µm was used to characterize the soil PSD. The soil samples, after removing the NOM, consisted of approximately 59% sand, 40% silt, and a very small percentage clay (i.e., < 1%), classifying it as sandy loam soil according to the standards set by the United States Department of Agriculture (USDA) soil taxonomy (Fig. SI-2a). The cumulative PSD of the spoil is shown in Fig. SI-2b. The measurements of soil acidity and wettability index, conducted following established protocols, indicated a *pH* of 6 and a hydrophilic wettability index of 43.5 dynes/cm.s^103–105^.

#### Removal of background NOM and potential MP

Even though soils with minimal background potential MP contamination were targeted for sampling, as a precautionary measure, soils were initially sieved using a 2-mm sized mesh to eliminate the possibility of large MP particles. It is noteworthy that no large plastic matter was detected during this process. We also removed the background NOM and the potential fraction of smaller MP particles in the soil by combusting it in a furnace at 570 °C for 3 h. The decomposition temperature of plastics is reported to range between 200 and 570 °C^106^. Thus, it was anticipated that any MP remnants in the soil would be eliminated^102^. Nevertheless, density separation and fluorescent microscopy were conducted following combustion to verify the removal of plastics from the soil samples. To do this, the protocol detailed in the cited references was followed^16,26^. Density separation was conducted twice using ~ 1.3 g*/*cm^3^ calcium chloride (CaCl_2_) solution. Fluorescence microscopy (Olympus BX43) was used to identify and quantify MP particles, employing a green light filter with excitation and emission wavelengths of 470 and 508 nm, respectively. Nile Red (NR) dye, prepared in n-hexane at a concentration of 50 mg/L, was applied for staining^107,108^. Finally, it confirmed the absence of remaining plastics in the soil samples.

#### Adding environmentally relevant MP and NOM to the base soil

A review of the literature indicates that the MP concentration of approximately 1–2% is commonly considered an environmentally relevant range for heavily contaminated agricultural and industrial soils^4,59,109^. However, this study examines a significantly lower range of 0.01% to enhance the generalizability of the findings. In addition to MP, we incorporated 3% NOM as dried peat moss (Fig. SI-3) residue to replace the NOM of the soil samples removed in the combustion process^110–112^. To prevent the introduction of any background MP while adding NOM, we conducted a thorough deionized water wash on the NOM sources before fragmenting them. The size of NOM residues was < 10 mm to reduce differences across soil samples and provide greater consistency in soil samples, as explained in the following section^113–115^.

MP particles and NOM were manually mixed with soil to provide a uniform distribution. To address the potential effect of MP particles and NOM distribution, following each test, soil samples were transferred to a steel tray, re-mixed thoroughly, and placed back in the container, and the experiment was repeated two more times. By doing so, we ensured that the same NOM and MP particles were used for all three duplicates but with a different distribution. The procedures outlined in the cited reference were followed for filling and saturating soil samples (Fig. SI-4), along with placing tensiometers^116,117^.

### Experimental setup (challenges and solutions)

**Fig. 1:**
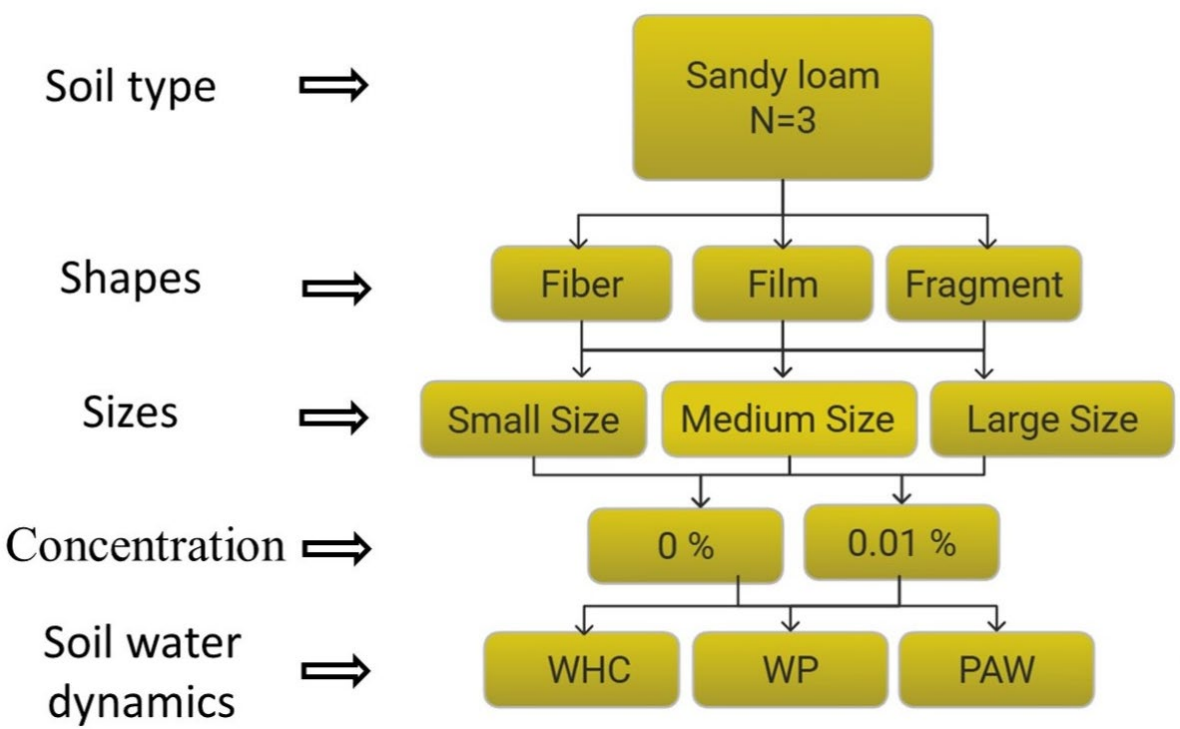
Schematic diagram of the experiment illustrating 10 contamination scenarios with variations in MP shape, size, and concentration (nine 0.01% contamination scenarios plus one clean 0% scenario). The number of duplicates for each scenario was N = 3. The small refers to the range sizes between 0.5 and 1 mm, medium refers to range sizes between 1 and 3 mm, and large refers to the range sizes between 3 and 5 mm.

As shown in Fig. 1, 10 contamination scenarios using HYPROP2 devices manufactured by METER Co. were investigated (Fig. SI-5). HYPROP2 utilize Schindler’s evaporation method to determine the unsaturated hydraulic characteristics of soil^118^. Changes in the soil–water dynamics of contaminated samples were compared with clean soil samples (controls). Each soil sample was comprised of 300 g of dry soil containing 3% *w/w* NOM, placed in a standard 250 cm^3^ stainless steel container^71,119^. Except for controls, all samples were contaminated with MP at 0.01%. Three different shapes of MP, i.e., film, fiber, and fragment, each characterized by three distinct size ranges, i.e., small (0.5–1 mm), medium (1–3 mm), and large (3–5 mm), were systematically introduced into the samples.

HYPROP2 was used to measure volumetric water content, unsaturated hydraulic conductivity, and soil matric potential in the range of 0 to − 100 kPa. HYPROP2 minimizes soil sample disturbance, significantly reduces data acquisition times, and allows measurement of a broader range of soil matric potentials^120,121^. Since the method relied on evaporation, the possibility of expediting the process was investigated by increasing the soil water evaporation rate using a 50-W infrared light source positioned 10 cm above the samples in a controlled environment with an electric dehumidifier as shown in Fig. SI-6. This adjustment reduced the time for each run to around 8 days. Another challenge addressed in this setup was the potential occurrence of power surges and outages during each run (~ 8 days). Experiments were compromised multiple times by this issue, resulting in the loss or incomplete termination of a multiday experiment; thus, an uninterruptible power supply (UPS) was utilized.

We conducted all experiments in a room with controlled environmental conditions, where temperature and humidity were continuously monitored to ensure minimal environmental variability and maintain consistent conditions throughout all tests. Recognizing the inherent uncertainties and potential errors in laboratory experiments, we mitigated these challenges by employing *N* = 3 duplicates for each experimental condition and applying statistical tools, as detailed below, to strengthen the reliability and robustness of our findings.

### Soil water retention curve (WRC) evaluation

WRC is commonly analyzed using WHC, WP, and PAW (Fig. SI-7) to assess soil water dynamics^72,73^. To determine WRC for each sample, the van Genuchten (VG) model was fitted to the volumetric water content, $\theta \; {\text{cm}}^{3}/{\text{cm}}^{3}$, and soil water matric potential, $\psi$ *-*kPa^122^. The VG model describes the relationship between $\theta$ and *ψ,* as:

1 $$\theta ={\theta }_{r}+\frac{\left({\theta }_{s}-{\theta }_{r}\right)}{{\left[1+{\left(\frac{\psi }{\alpha }\right)}^{n}\right]}^{\left(\frac{1}{m}\right)}}$$

where, ${\theta }_{r}$ ${\text{cm}}^{3}$/${\text{cm}}^{3}$ is the residual water content, ${\theta }_{s} \; {\text{cm}}^{3}/{\text{cm}}^{3}$ is the saturated water content, α *1/*cm is a parameter corresponding to the inverse of the air-entry value, and *n* and $m=1-1/n$ (−) are dimensionless fitting parameters^123–125^.

As in many studies, WHC was determined by identifying the matric potential of − 33 kPa, representing the condition at which soil retain water against gravity and for root uptake^72,126^. WP was determined by identifying the point in the WRC where the curve transitions to a nearly flat shape, indicating the moisture level at which plants can no longer extract water, leading to permanent wilting (Fig. SI-7)^73,127,128^. PAW, as a crucial characteristic of soil, was defined as the difference between WHC and WP moisture levels^72^.

### Statistical analysis

We analyzed the data to assess statistically significant differences between the control group and the contaminated groups. Welch’s t-test, a modification of the Student’s t-test suitable for unequal variances, was employed to compare the means of each treatment group to the control group^129–131^. For each comparison, we estimated the *p*-value and the 95% confidence interval (CI) of the true difference in means^132,133^. Additionally, we verified that the data within each group followed a normal distribution, thereby justifying the use of the t-test^134^. This rigorous database and statistical approach ensured that our comparisons were valid and reliable, providing a robust assessment of the differences between measured data sets.

## Results

**Fig. 2:**
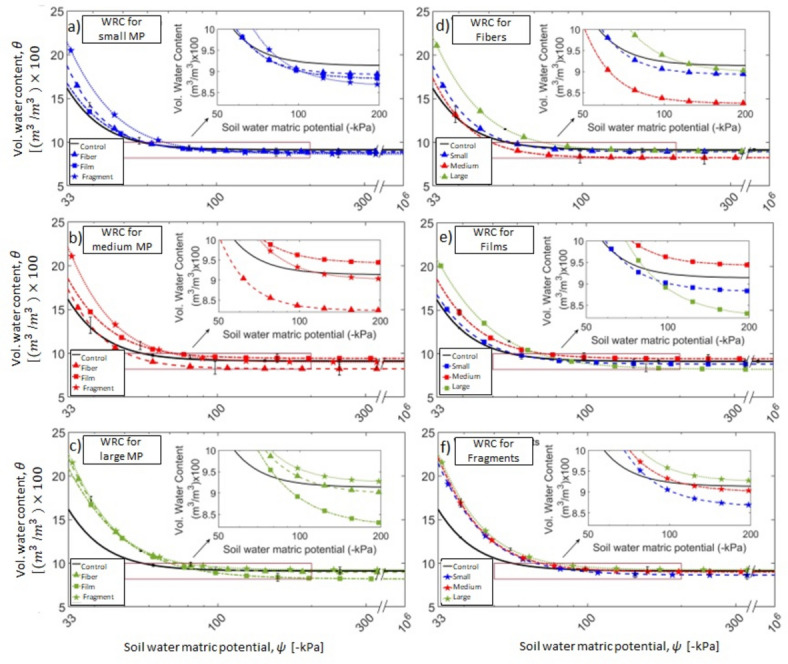
Soil Water Retention Curves in combined size-shape format: (**a**) represents all shapes with the range of 0.5–1 mm (small); (**b**) represents all shapes with the range of 1–3 mm (medium); (**c**) represents all shapes with the range of 3–5 mm (large); (**d**) represents all sizes of fibers; (**e**) represents all sizes of films; and, (**f**) represents all sizes of fragments.

**Fig. 3:**
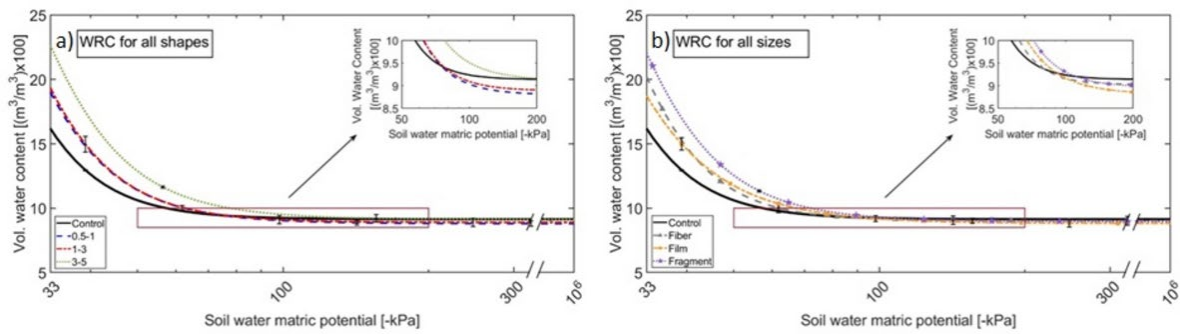
Soil Water Retention Curves between: (**a**) is plotted in size format. Blue, red, and green lines refer to average values of all sizes. (**b**) is plotted in the shape format. Gray, orange, and purple lines refer to all shapes. The small means 0.5 and 1 mm, medium refers to range sizes between 1 and 3 mm, and large refers to the range sizes between 3 and 5 mm.

To clearly convey meaningful information from the data collected, data are presented in three different formats, each using a different color and a symbol code (see Table SI-1). Color and symbol codes have been used consistently in the entire article: black and circle, grey and triangle, orange and square, and purple and star denote control, fiber, film, and fragments, respectively. All data within this section are classified into three distinct figure and table formats: 1) size format, 2) shape format, and 3) combined size-shape format. In the size format, all shapes are averaged and categorized only by their sizes. The combined shape-size format includes data segregated according to both the size and shape of MP (e.g., as depicted in Fig. 2a–f). Figure 3a illustrates the WRC in size format. For example, data assigned to the small MP (0.5–1 mm) includes an average value of MP particles with 0.5–1 mm size of all shapes, i.e., fiber, film, and fragment. Figure 3b illustrates the WRC in shape format. For example, data assigned to fiber in Fig. 3b includes an average value of fiber MP particles with all sizes, i.e., small, medium, and large. From these WRCs, key parameters such as WHC, WP, and PAW are derived, as discussed in the following sections. All raw data is available in the SI section.

### Water holding capacity (WHC)

**Fig. 4:**
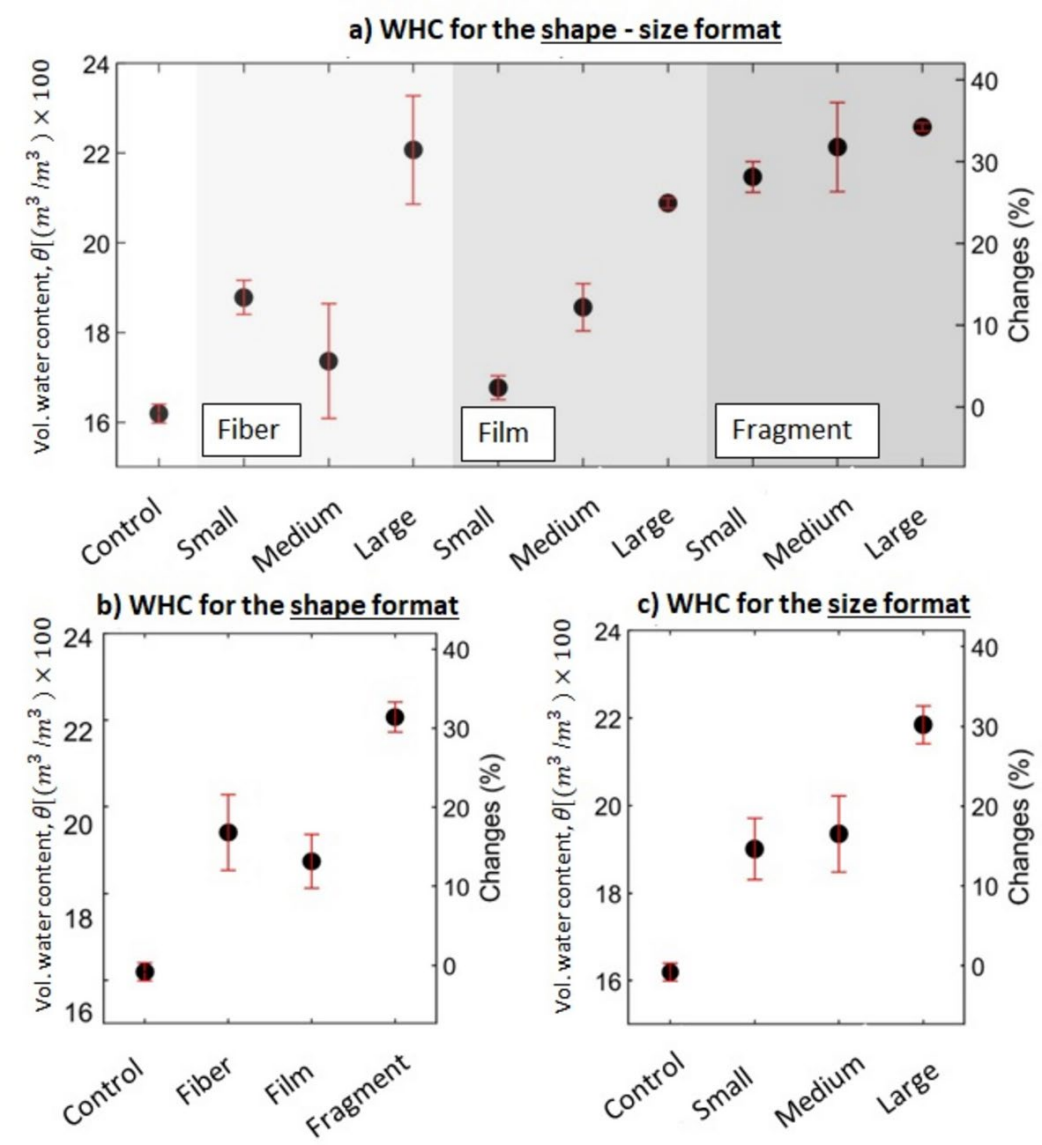
Changes in the WHC for the (**a**) shape-size format, (**b**) shape format, and (**c**) size format. The small refers to the range sizes between 0.5 and 1 mm, medium refers to range sizes between 1 and 3 mm, and large refers to the range sizes between 3 and 5 mm.

WHC data are presented in combined shape-size format in Fig. 4a and Table SI-2, in which each point represents an average value, including its CI^133^. The addition of MP to clean soil increased the WHC in all scenarios (Fig. 4a). Fragments exhibited the most significant change in WHC followed by fibers and films. Table SI-2 and Fig. 4b represent shape format and reveal that the average value of WHC increases with the addition of MP particles, from 0.162 cm^3^*/*cm^3^ for the control by 19.8%, 15.7%, and 36.3%, for fiber, film, and fragment, respectively. Comparing WHC of fiber, film, and fragment against control led to *p*-values of 0.01, 0.01, and < 0.01, respectively. These suggest a statistically significant impact on WHC of all shapes (Fig. 4b). Additionally, the CI of fiber is larger than film and fragment, implying a greater variability and uncertainty in the influence of size factor in fibers, followed by film and fragment.

Table SI-2 and Fig. 4c represent results in a size format. MP with sizes between small, medium, and large MP particles can increase the average WHC from 0.162 cm^3^*/*cm^3^ for the control by 17.4%, 19.5%, and 34.9%, respectively. Comparing WHC of all size categories against control led to *p*-values of 0.01 for each of them. This suggests a statistically significant impact on WHC of all sizes (Fig. 4c). Additionally, the CI of large MP is less than small and medium MP implying less variability and uncertainty in the influence of shape factor in the largest size category.

Previous studies have demonstrated that spatial variations in wettability and aggregate distribution, caused by the presence of compounds with differing hydrophilicity in soil mixtures (in this study MP particles), can influence key physical processes such as infiltration, preferential flow, and the distribution and movement of soil moisture^96,97,135,136^. Thus, it can be hypothesized that all MP particles, particularly fragment shapes (Fig. 4b) and large MP particles (Fig. 4c), enhance the attractive forces responsible for holding water against gravity^137^. This can be attributed to the increase in total micropores and the adhesion forces between water molecules and weak hydrophilic MP particles^47,138^. To elucidate how MP particles influence soil micro- and macropore structure, and therefore WHC, advanced imaging techniques such as Computed Tomography and X-ray could provide useful information that is not the subject of this study. However, we direct interested readers to the cited articles for detailed discussions on applying these imaging techniques in studying soil pore structure^139–143^.

### Wilting point (WP)

**Fig. 5:**
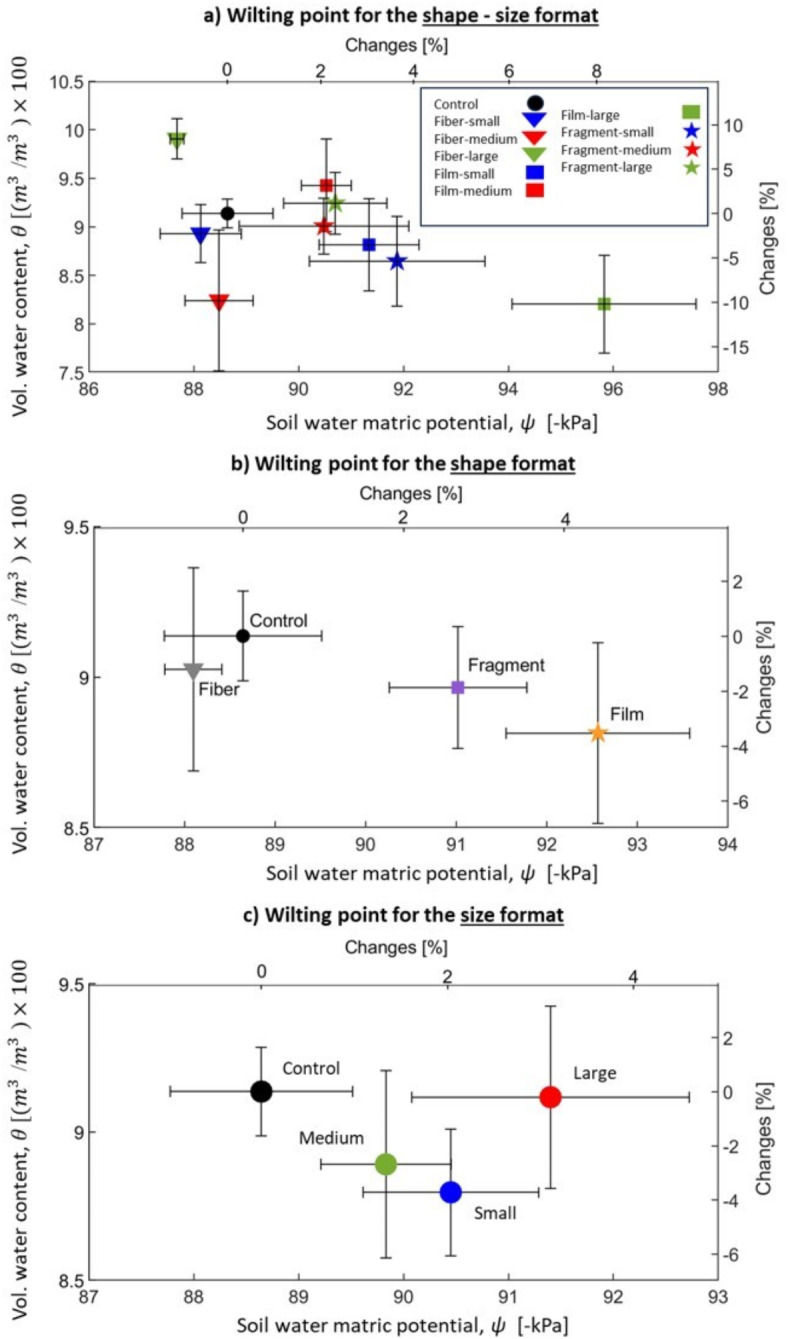
Wilting points changes for various size ranges and shapes of microplastics compared to the control. The small refers to the range sizes between 0.5 and 1 mm, medium refers to range sizes between 1 and 3 mm, and large refers to the range sizes between 3 and 5 mm.

Unlike WHC, which was inherently linked to − 33 kPa pressure (standardized value in the soil literature), the WP can be characterized by wilting point water content (WP-$\theta ),$ and wilting point matric potential (WP-$\psi$), which are presented in the combined shape-size format, shape format, and size format in Fig. 5a–c, respectively^73^.

Table SI-3 and Fig. 5b present shape format and reveal that the average value of WP-*θ* slightly decreases with the addition of MP particles, from 9.1 at the control to − 0.8%, − 3.1%, and − 1.4% for fiber, film, and fragment, respectively. Comparing the WP-*θ* of all shapes against the control led to *p*-values of 0.78, 0.36, and 0.51 for fiber, film, and fragment, respectively. This suggests a statistically insignificant impact on WHC of all sizes (Fig. 4c). Additionally, the CI for fiber and film are relatively wider compared to that of fragments, implying greater variability and uncertainty in the influence of sizes within the fiber and film category, highlighting the importance of size variations within these classifications (Fig. 5a). This observation is consistent with the resulting impact on WHC and may be attributed to the physical flexibility of fiber and film through the pore spaces.

Table SI-3 and Fig. 5b reveal that the average WP-ψ slightly decreases for film and fragment from − 88.6 kPa for controls by − 4.4% and − 2.7%, respectively. Meanwhile, the average WP-ψ increases for fibers by 0.6%. Comparing WP-ψ of fragment, film, and fiber against control led to *p*-values of 0.09, 0.02, and 0.59, respectively. These suggest a statistically insignificant impact on WP-ψ of fragment and fiber, compared to that of film that is statistically significant (Fig. 5b). The disruption of thin water films within soil micropores at the wilting point, commonly referred to as capillary breaks, plays a critical role in altering WP-ψ. These water films are essential for sustaining hydraulic connectivity among retained water molecules, and their breakdown significantly impacts soil ability to transport water, particularly under extreme moisture stress conditions^144^. Thus, it can be hypothesized that the planar and flexible nature of film MP particles may serve as a more effective barrier within the soil matrix, disrupting the connectivity of micropores. This disruption can result in stronger capillary breaks, ultimately causing an increase in WP-ψ. In contrast, fibers and solid fragments seem to behave differently, potentially exerting less barrier effects and less influence on the capillary break mechanism. Additionally, the CI for fragment and film are relatively wider than that of fiber, implying greater variability and uncertainty in the influence of sizes within the fragment and film category.

Table SI-3 and Fig. 5c represent results in a size format. MP with sizes between small, medium, and large MP particles can decrease the average WP-θ from 9.1 for the control by 3.3%, 2.3%, and 0.2%, respectively. Comparing WP-θ of small, medium, and large MP against control led to *p*-values of 0.23, 0.50, and 0.98, respectively. These suggest a statistically insignificant impact on WP-θ of all sizes (Fig. 5c). Additionally, the CI relatively increases from smallest to largest sizes, implying an increase in variability and uncertainty in the influence of shape factor from smallest to largest size categories.

Table SI-3 and Fig. 5c reveal that the average WP-ψ decreases from − 88.6 kPa for the control group by − 2.0%, − 1.3%, and − 3.1% for small, medium, and large MP, respectively. Comparing WP-ψ of small, medium, and large MP against control led to *p*-values of 0.15, 0.33, and 0.11, respectively. These suggest a statistically insignificant impact on WP-ψ of all sizes (Fig. 5c). It can be hypothesized that larger MP may have a greater influence on altering the capillary break mechanism due to their size. Additionally, the CI of large MP is relatively large, implying great variability and uncertainty in the influence of the shape factor for this size category.

### Plant available water (PAW)

**Fig. 6:**
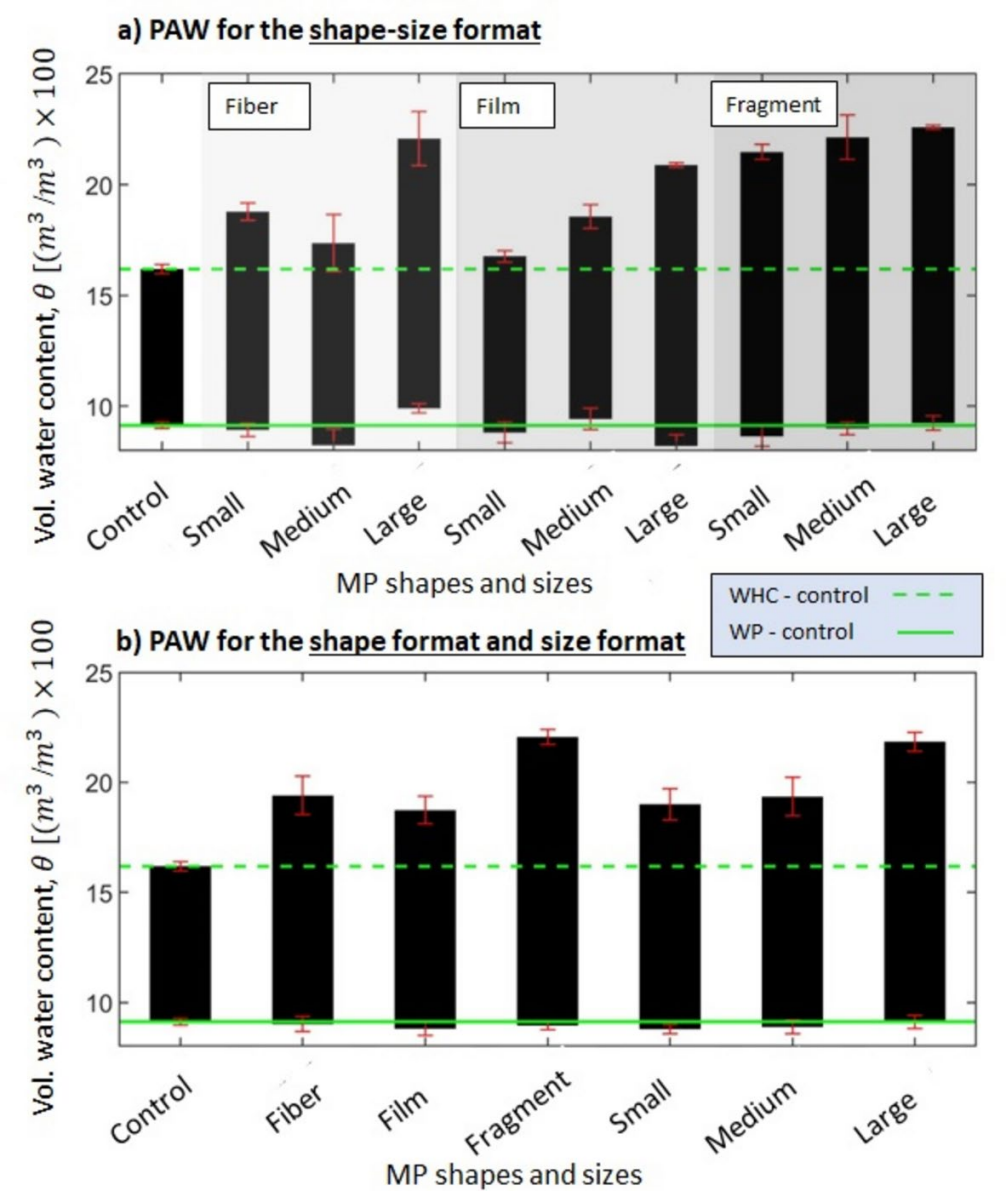
Plant available water for the (**a**) shape-size format, (**b**) shape format and the size format. The small refers to the range sizes between 0.5 and 1 mm, medium refers to the range sizes between 1 and 3 mm, and large refers to the range sizes between 3 and 5 mm.

A combined shape-size format (Fig. 6a) is presented alongside corresponding quantitative information (Table SI-4). Each bar in Fig. 6a,b represent PAW, with the top and bottom denoting average WHC and WP, respectively. In Fig. 6, the solid lines represent the average WP, and dashed lines represent the average WHC of clean control samples. Error bars depict standard errors of WHC and associated WP. The addition of MP particles to clean soil increases the WHC in almost all scenarios and has a minor impact on WP, as discussed earlier. As shown in Fig. 6b and Table SI-4, fragments exhibit the most significant increase in PAW, 85.7%, followed by fibers and films, with 47.1% and 40.7% increases, respectively. Results also show that large MP particles, show the most significant increase in PAW, 80.5%, followed by small and medium MP, with 44.7% and 48.3% increases, respectively.

## Discussion

This study shows that the presence of even a small amount of MP particles—just 0.01% of the soil mass—can significantly affect the ability of agricultural sandy loam soils in retaining and releasing moisture. The impact is especially affected by the morphological characteristics of MP particles (i.e., their shapes and sizes). The results demonstrated that MP contamination at a concentration of 0.01% can have a statistically significant impact on the soil WHC and total PAW, while its effect on WP was statistically insignificant. However, it should be noted that although increasing WHC and PAW generally benefits farmers by increasing crop yields, enabling crop diversification, and reducing farming-related stress through better irrigation, it can also have adverse effects. Among these are waterlogging, an increase in pest and disease outbreaks, root decay, and a reduction in soil oxygen levels. Such issues can significantly impact plant health and soil quality. Additionally, groundwater recharge patterns can be affected by an increase in PAW on a larger scale. It may increase evapotranspiration and soil–water evaporation. In the long run, these changes can have domino effects on the watershed hydrology. Our results show that MP contamination at 0.01% has minor impacts on WP-ψ and WP-θ, suggesting minimal effects on plants with strong water potential (e.g., < − 400 kPa)^145^. However, less water-resistant plants may be more affected by soil-MP pollution.

Moreover, results indicate that the impact of MP size on WHC was consistent across fragment and film shapes, with larger particle sizes leading to a greater effect (increase) on WHC. This suggests that as the size of films and fragments increases, so does their ability to disrupt gravitational drainage and, accordingly, PAW. However, this trend was not observed for fibers, likely due to their distinct physical properties. Fibers possess a high capacity for bending and tangling within the soil matrix, which may limit their ability to influence WHC in the same manner as fragments and films. Furthermore, no clear relationship between particle size and WP-ψ or WP-θ was observed, aligning with the conclusion that the impacts are relatively minor. As noted in previous studies, soil pore structure and connectivity, along with solid particle hydrophilicity and water attachment forces, are key factors influencing soil hydrological properties such as water retention and movement. Our results suggest that MP particles of different shapes and sizes may have varying impacts on these factors. However, to directly observe and measure these impacts, advanced imaging techniques such as micro-computed tomography or X-ray imaging are required.

This study offers valuable insights that could be used to improve applied agricultural and soil management practices. Because of the prevalence of MP in soils and the likelihood of increasing contamination due to continued plastic use, it is essential to prepare for and mitigate these impacts. Understanding how MP particles affect soil water retention may enable future agricultural systems to adjust irrigation schedules, enhancing water efficiency and growth productivity. This study shows that the impacts of MP on PAW can be significant in specific circumstances that can affect crop yields and, on a larger scale, regional food security. Increased irrigation demands due to altered water dynamics could exacerbate water scarcity. Consequently, these changes also affect the health of soils, microbial activity, and carbon sequestration, further stressing ecosystems.

Given that biosolids broadly used as fertilizers contain high MP concentrations, the findings of this study reinforce the importance of stricter waste management regulations to limit MP accumulation in agricultural soils. Moreover, these insights support sustainable plastic use policies, encouraging the adoption of biodegradable alternatives and improved waste disposal practices to mitigate MP contamination. Decision-makers can also implement strategies to extract or digest MP particles within wastewater treatment plants before biosolid application, reducing MP pollution in agricultural lands.

This study serves as an initial demonstration, highlighting the significant impact of MP on PAW under certain conditions. However, limitations remain regarding the sensitivity of different soil and plant types to MP pollution, and the diverse shapes and forms of MP in real-world scenarios, warranting further investigations. The use of robust statistical methods and duplicate experiments would enhance the reliability and generalizability of future findings. Although scaling up to field ecosystems introduces complexities due to variations in soil structure, MP distribution, climate conditions, and biotic interactions, it can lead to more practical soil MP pollution solutions. Overall, this study provides valuable insight into soil–water dynamics, but future studies will need to take into account the complexity of real-world conditions and improve their applicability to diverse agricultural systems.

## Supplementary Information

Supplementary Information.

## Data Availability

All raw data are available in the supplementary information (SI) section in .xls format. Several supporting tables and plots are also available in .doc format. All data can also be provided upon request from FJ, the corresponding author, through his email address, fjazaei@memphis.edu.
