# Supplementary material for: Potential impacts of microplastic pollution on soil–water–plant dynamics
Source: Sci Rep. 2025 Mar 21;15:9784. doi: 10.1038/s41598-025-93668-0 (PMC11928583; doi:10.1038/s41598-025-93668-0)
Supplement: Supplementary file 1 — Supplementary Information. [file 41598_2025_93668_MOESM1_ESM.docx]

**Supplementary Information**

# **Potential impacts of microplastic pollution on soil-water-plant dynamics**

Alireza Bakhshaee^a^, Peyman Babakhani^b^, Muhammad Masood Ashiq^a^, Kati Bell^c^, Maryam Salehi^d^, Farhad Jazaei^a,e,*^

a Department of Civil Engineering, University of Memphis, Memphis, TN, USA

b Ground Engineering, University of Manchester, Manchester, UK

c Research and Innovation, Brown and Caldwell, Nashville, TN, USA

d Department of Civil and Environmental Engineering, University of Missouri, Columbia, MO, USA

e Center for Applied Earth Science and Engineering Research, University of Memphis, Memphis, TN, USA

* Corresponding author: [fjazaei@memphis.edu](mailto:fjazaei@memphis.edu)


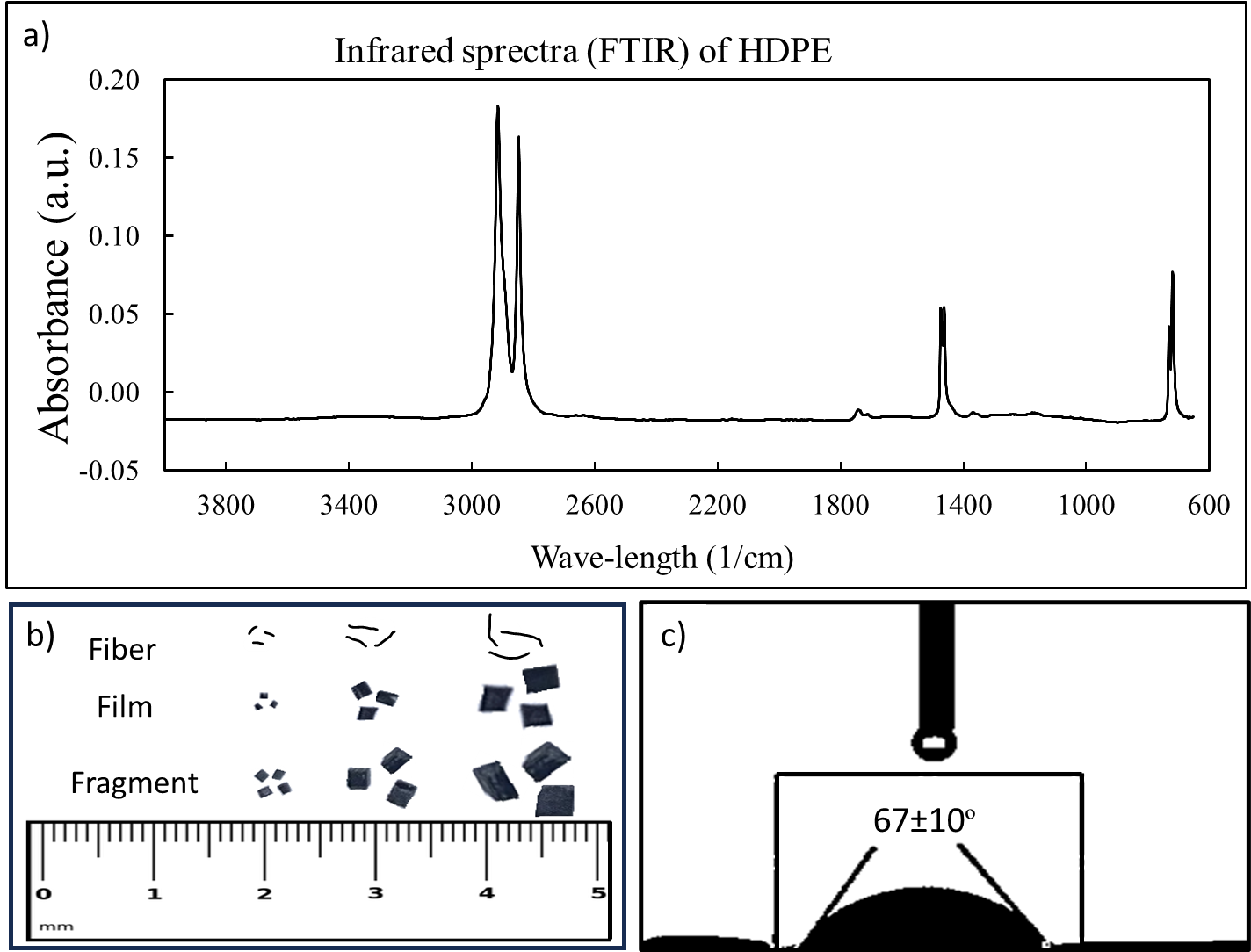


***Fig. SI-1*** *Different shapes and sizes of HDPE MP were used in this study: a) FTIR spectroscopy results for the HDPE. HDPE typically exhibits distinct peaks around 2918* ${cm}^{-1}$ *(symmetric* ${CH}_{2}$ *stretching) and 2848* ${cm}^{-1}$ *(asymmetric* ${CH}_{2}$ *stretching) due to the presence of hydrocarbon chains* ^85^. *Additionally, peaks around 1460* ${cm}^{-1}$ *(*${CH}_{2}$ *bending) further confirm the presence of HDPE* ^86^. *b) three range sizes and shapes of MPs and c) water contact angle of MP used in this study. On a weakly hydrophilic surface, the water droplet does not spread completely but will appear slightly flattened, with a larger contact angle (>30°) than on a strongly hydrophilic surface (which can be close to 0°)* ^71,89^*. However, a droplet does not bead up tightly like a very hydrophobic surface (with a contact angle greater than 90°).*


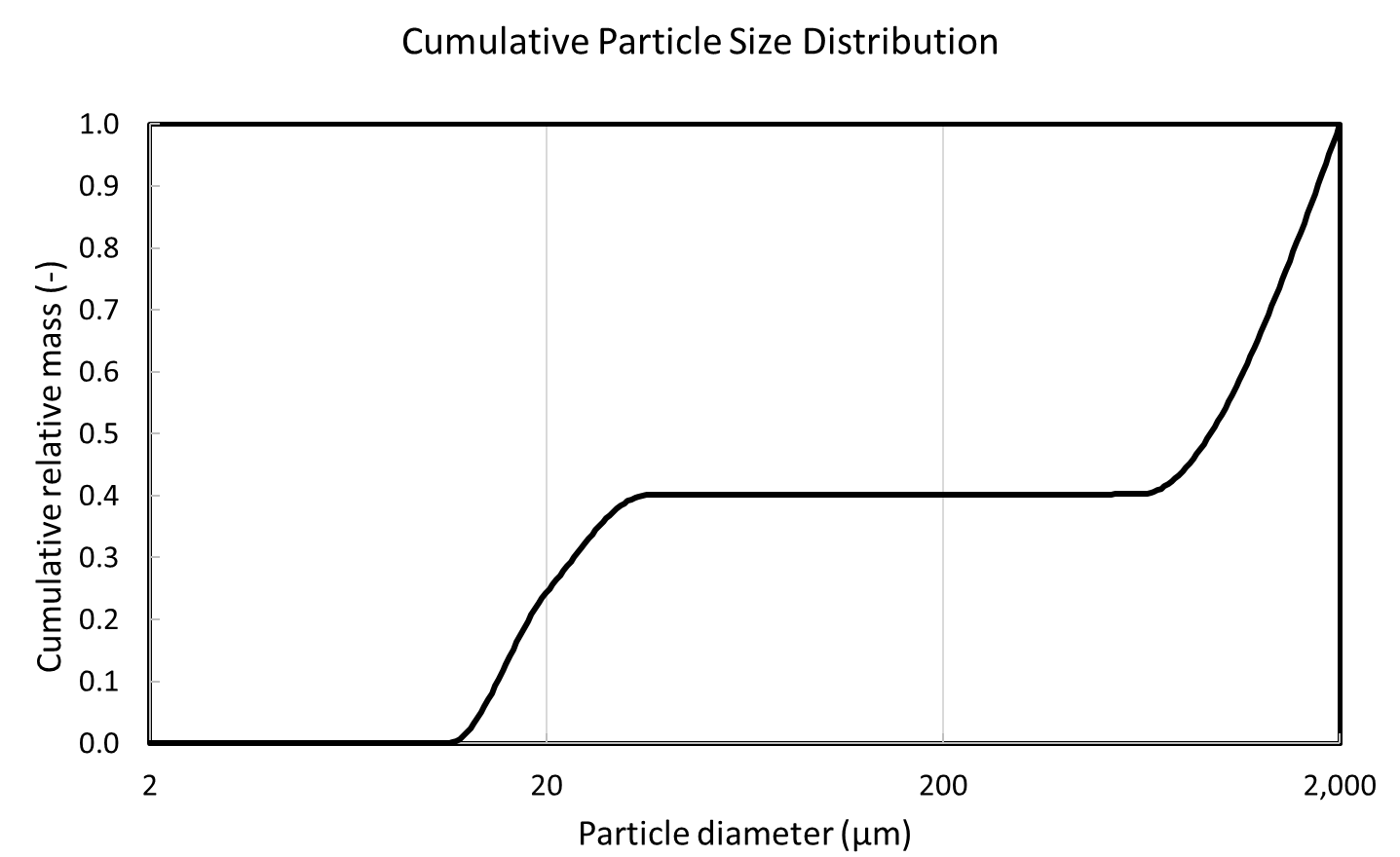


**b)**

***Fig. SI-2*** *a) US soil taxonomy came from PARIO. b) Sandy loam cumulative particle size distribution*


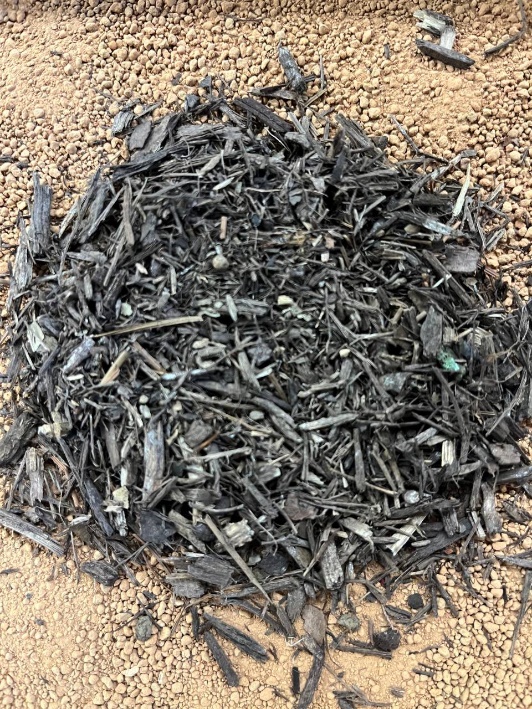


***Fig. SI-3*** *Peat moss added to the soil.*


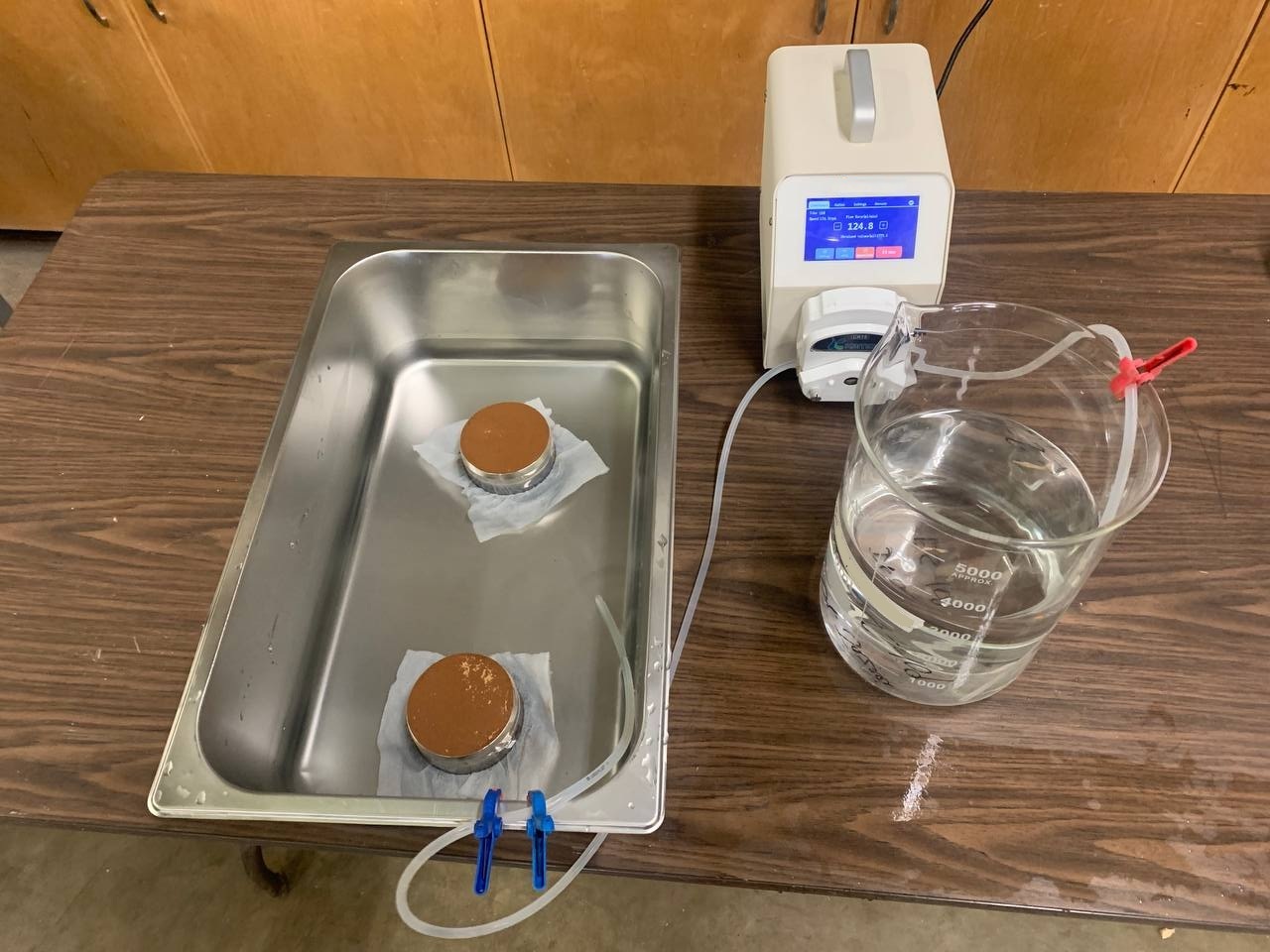


***Fig. SI-4*** *Saturating the soil by putting the soil sample into the tray and raising the water level up to 5 mm below the top of the sample rim.*

*
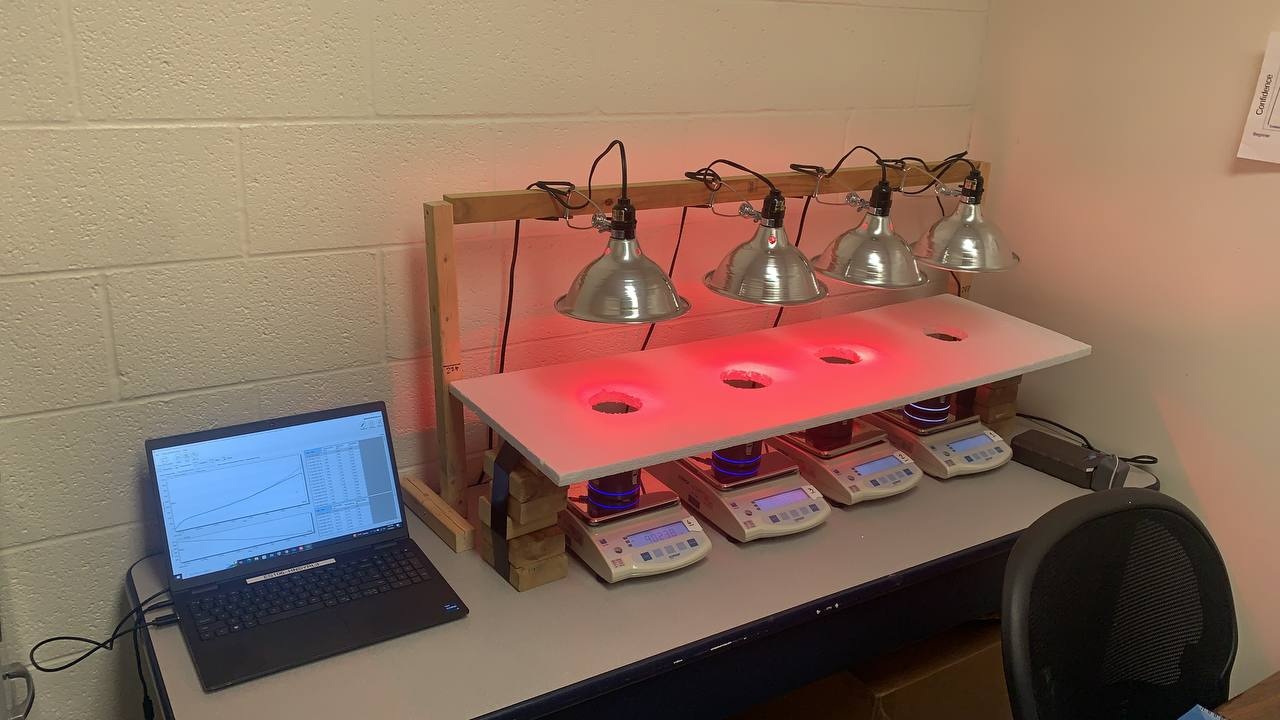
*

***Fig. SI-5*** *Laboratory experiment setup*


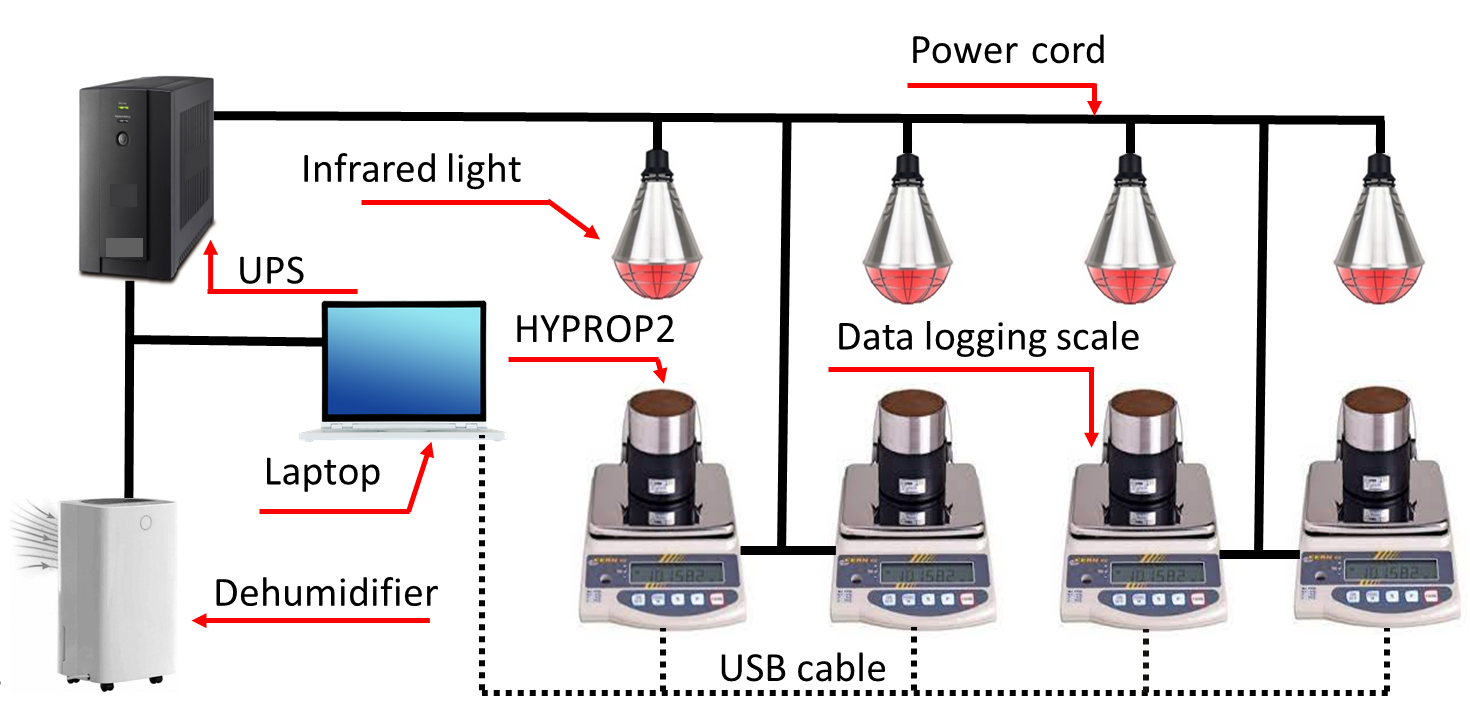


***Fig. SI-6*** *Schematic diagram of the laboratory experiment setup. It includes scales and HYPROP2 devices connected via a universal serial bus (USB) to a laptop for real-time data logging. All electrical components—dehumidifier, laptop, and infrared lights—are powered through an uninterruptible power supply (UPS), ensuring continuous operation during power outages.*


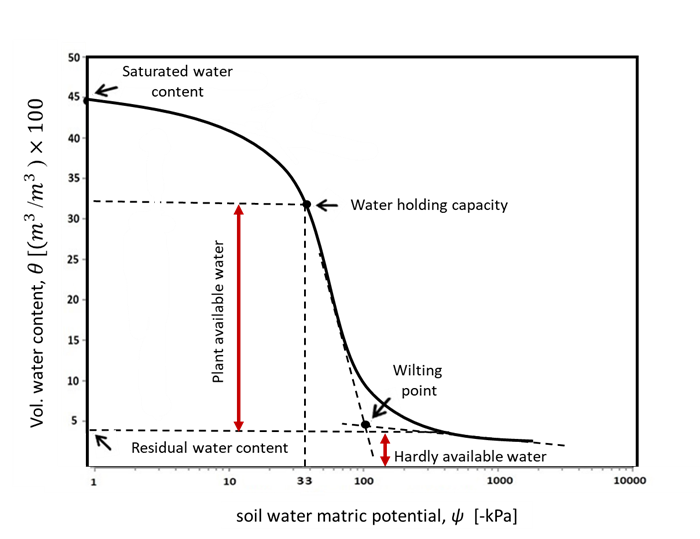


***Fig. SI-7*** *The soil water retention curve shows the wilting point, field capacity, available water for plants, and saturated water content.*

***Table. SI-1*** *Color and symbol codes for various shapes of MP and control, used to represent data throughout the article.*

| Shape | Color | Symbol |
| --- | --- | --- |
| Control | Black |  |
| Fiber | Grey |  |
| Film | Orange |  |
| Fragment | Purple |  |

***Table. SI-2*** *Changes in the water content of the water holding capacity.*

| Shapes | Size range [mm] | | WHC [$\frac{{cm}^{3}}{cm^{3}}\times100$] | | Changes in WHC (%) | | p-value |
| --- | --- | --- | --- | --- | --- | --- | --- |
| Control | 0 | 16.2 ± 0.2 | | 0 | | - | |
| Fiber | 0.5 – 1 | 18.8 ± 0.4 | | 16.0 ± 2.5 | | - | |
|  | 1 – 3 | 17.4 ± 1.3 | | 7.2 ± 8.2 | | - | |
|  | 3 – 5 | 22.1 ±1.2 | | 36.3 ± 7.5 | | - | |
| Film | 0.5 – 1 | 16.8 ± 0.3 | | 3.6 ± 2 | | - | |
|  | 1 – 3 | 18.6 ± 0.5 | | 14.6 ±3.3 | | - | |
|  | 3 – 5 | 20.9 ± 0.1 | | 29.0 ± 0.6 | | - | |
| Fragment | 0.5 – 1 | 21.5 ± 0.3 | | 32.6 ± 2 | | - | |
|  | 1 – 3 | 22.1 ± 1.0 | | 36.7 ± 5.9 | | - | |
|  | 3 – 5 | 22.6 ± 0.1 | | 39.5 ± 0.6 | | - | |
| Fiber | | 19.4 ± 0.9 | | 19.8 ± 5.5 | | 0.01 | |
| Film | | 18.7 ± 0.6 | | 15.7 ± 3.4 | | 0.01 | |
| Fragment | | 22.1 ± 0.3 | | 36.3 ± 2 | | <0.01 | |
| 0.5 – 1 mm | | 19.0 ± 0.7 | | 17.4 ± 4.2 | | 0.01 | |
| 1 – 3 mm | | 19.3 ± 0.8 | | 19.5 ± 4.6 | | 0.01 | |
| 3 – 5 mm | | 21.8 ± 0.4 | | 34.9 ± 2.1 | | 0.01 | |

***Table. SI-3*** *Changes in water content and pressure at the wilting point.*

| Shapes | Size range [mm] | WP-θ $[({cm}^{3}/{cm}^{3})$  $\times100]$ | Change of volumetric water content [%] | p-value  WP-θ | WP-ψ  [-kPa] | Change of WP-ψ  [%] | p-value  WP-ψ |
| --- | --- | --- | --- | --- | --- | --- | --- |
| Control | 0 | 9.1 ± 0.1 | 0 | - | 88.6 ± 0.9 | 0 | - |
| Fiber | 0.5 – 1 | 8.9 ± 0.3 | -1.9 ± 0.5 | - | 88.1 ± 0.8 | 0.6 ± 0.3 | - |
|  | 1 – 3 | 8.2 ± 0.7 | -9.4 ± 8 | - | 88.5 ± 0.6 | 0.2 ± 0.7 | - |
|  | 3 – 5 | 9.9 ± 0.2 | 8.9 ± 2.3 | - | 87.7 ± 0.1 | 1.1 ± 0.1 | - |
| Film | 0.5 – 1 | 8.8 ± 0.5 | -3.1 ± 5.2 | - | 91.3 ± 0.9 | -3.0 ± 1.1 | - |
|  | 1 – 3 | 9.4 ± 0.5 | 3.6 ± 5.2 | - | 90.5 ± 0.5 | -2.1 ± 0.5 | - |
|  | 3 – 5 | 8.2 ± 0.5 | -9.8 ± 5.5 | - | 95.8 ± 1.8 | -8.1 ± 2.0 | - |
| Fragment | 0.5 – 1 | 8.6 ± 0.5 | -4.9 ± 5.1 | - | 91.9 ± 1.7 | -3.6 ± 1.9 | - |
|  | 1 – 3 | 9.0 ± 0.3 | -1.0 ± 3.2 | - | 90.5 ± 1.6 | -2.1 ± 1.8 | - |
|  | 3 – 5 | 9.2 ± 0.3 | 1.6 ± 3.5 | - | 90.7 ± 1.0 | -2.3 ± 1.1 | - |
| Fiber | | 9.0 ± 0.3 | -0.8 ± 3.7 | 0.78 | 88.1 ± 0.3 | 0.6 ± 0.4 | 0.59 |
| Film | | 8.8 ± 0.3 | -3.1 ± 3.3 | 0.36 | 92.6 ± 1.0 | -4.4 ± 1.1 | 0.02 |
| Fragment | | 8.9 ± 0.2 | -1.4 ± 2.2 | 0.51 | 91.0 ± 0.8 | -2.7 ± 0.9 | 0.09 |
| 0.5 – 1 mm | | 8.8 ± 0.2 | -3.3 ± 2.4 | 0.23 | 90.4 ± 0.8 | -2.0 ± 0.8 | 0.15 |
| 1 – 3 mm | | 8.9 ± 0.3 | -2.3 ± 3.5 | 0.50 | 89.8 ± 0.6 | -1.3 ± 0.7 | 0.33 |
| 3 – 5 mm | | 9.1 ± 0.3 | -0.2 ± 3.4 | 0.98 | 91.4 ± 1.3 | -3.1 ± 1.5 | 0.11 |

***Table. SI-4*** *Changes in plant available water.*

| Shapes | Size range [mm] | PAW  $[({cm}^{3}/{cm}^{3})$  $\times100]$ | Change of PAW [%] |
| --- | --- | --- | --- |
| Control | 0 | 7.1 ± 0.4 | 0 |
| Fiber | 0.5 – 1 | 9.8 ± 0.7 | 39.6 ± 9.6 |
|  | 1 – 3 | 9.1 ± 2.0 | 29.3 ± 28.5 |
|  | 3 – 5 | 12.2 ± 1.4 | 72.5 ± 20.1 |
| Film | 0.5 – 1 | 8.0 ± 0.7 | 12.8 ± 10.5 |
|  | 1 – 3 | 9.1 ± 1.0 | 29.5 ± 14.2 |
|  | 3 – 5 | 12.2 ± 0.6 | 79.8 ± 8.7 |
| Fragment | 0.5 – 1 | 12.8 ± 0.8 | 81.8 ± 11.3 |
|  | 1 – 3 | 13.1 ± 1.3 | 86.1 ± 18.2 |
|  | 3 – 5 | 13.3 ± 0.4 | 89.1 ± 5.8 |
| Fiber | | 10.4 ± 1.2 | 47.1 ± 17.1 |
| Film | | 9.9 ± 0.9 | 40.7 ± 13.1 |
| Fragment | | 13.1 ± 0.5 | 85.7 ± 7.8 |
| 0.5 – 1 mm | | 10.2 ± 0.9 | 44.7 ± 13.0 |
| 1 – 3 mm | | 10.5 ± 1.2 | 48.3 ± 16.8 |
| 3 – 5 mm | | 12.7 ± 0.7 | 80.5 ± 10.5 |


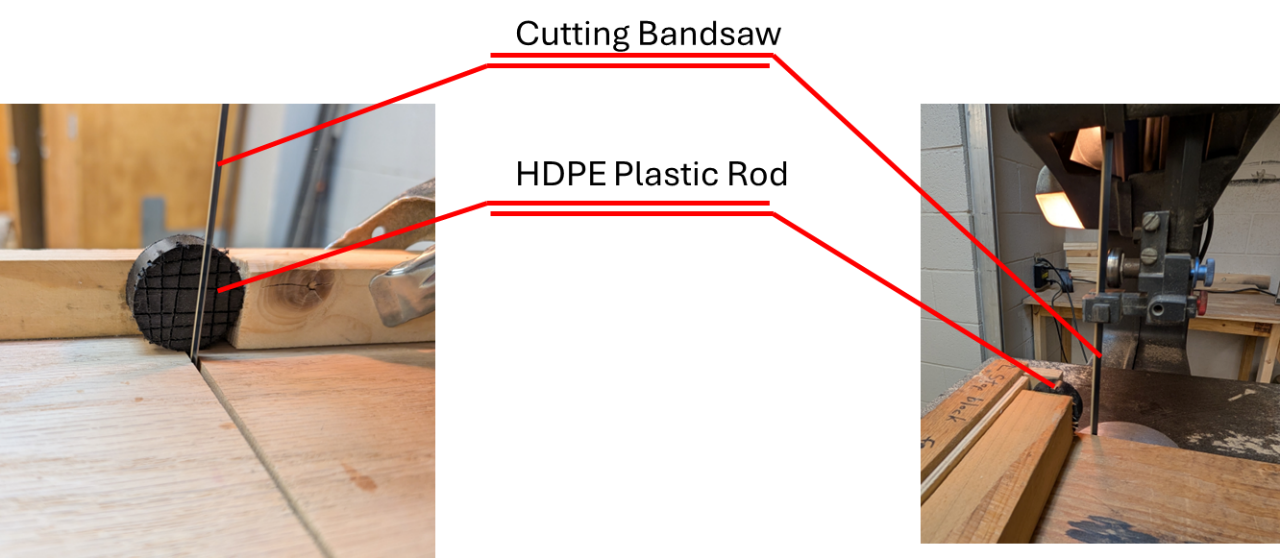


***Fig. SI-8*** *Cutting plastic rods to produce fragment MPs using a Bandsaw machine.*

*
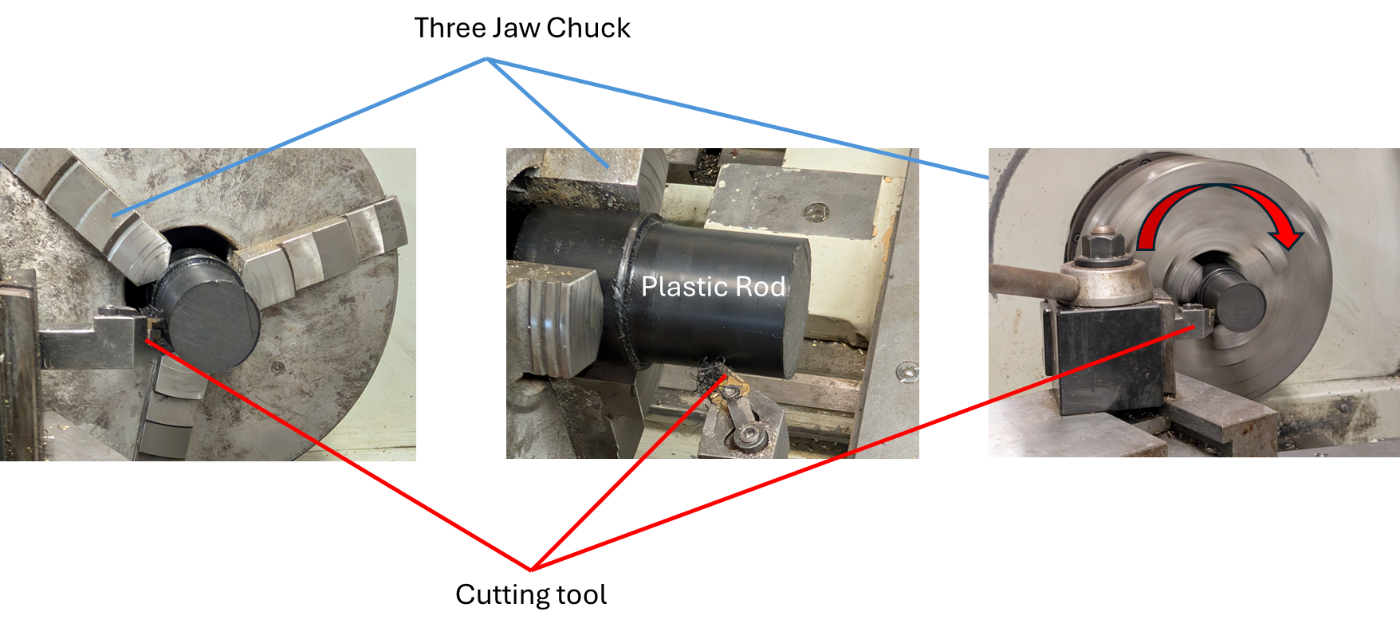
*

***Fig. SI-9*** *Cutting plastic rods to produce film and fiber MP particles using a Swing Metal Lathes.*


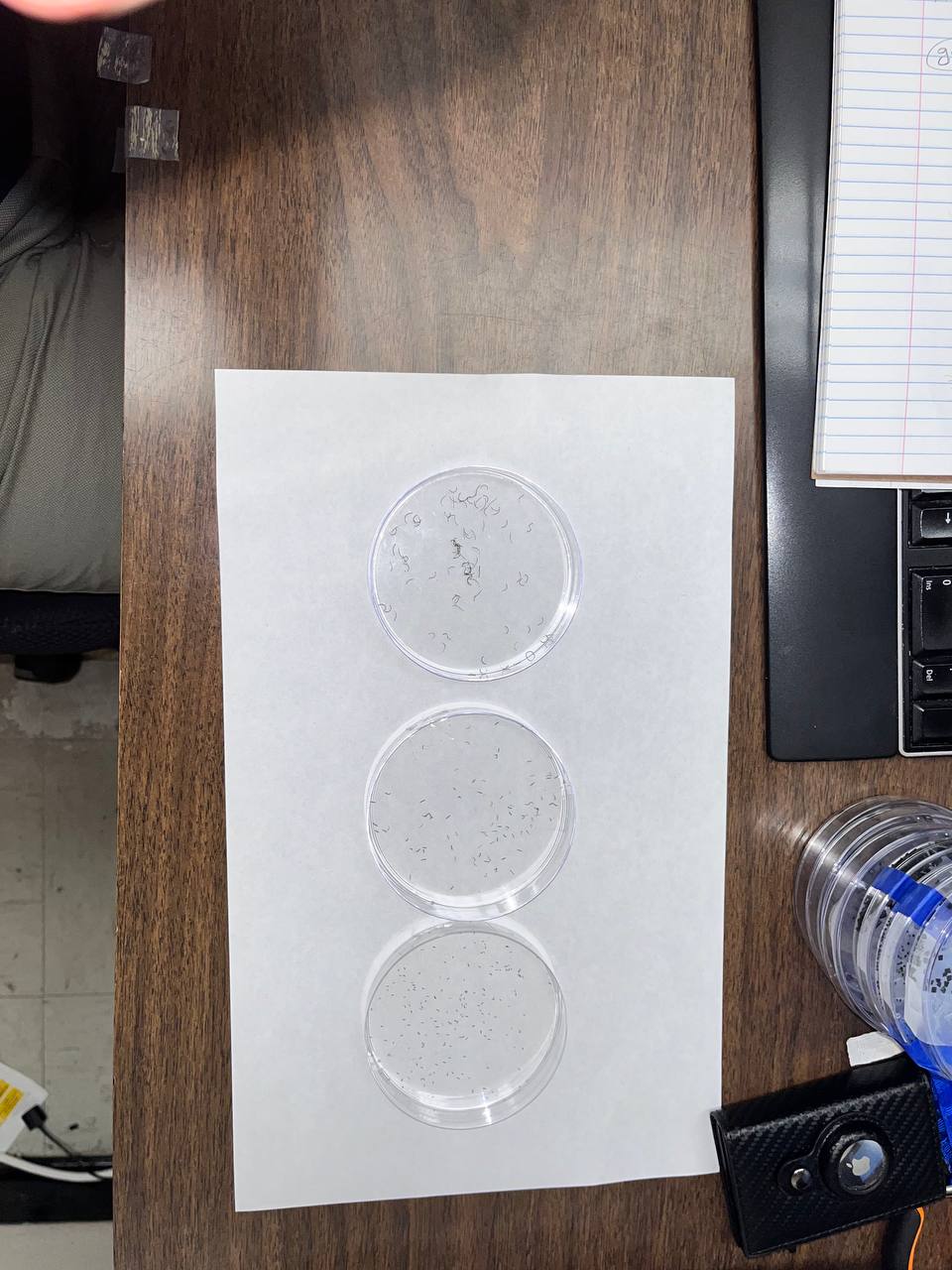


***Fig. SI-10*** *Fibers for adding into the soil. From left to right, the fibers are sized 0.5 - 1 mm, 1 - 3 mm, and 3 - 5 mm.*


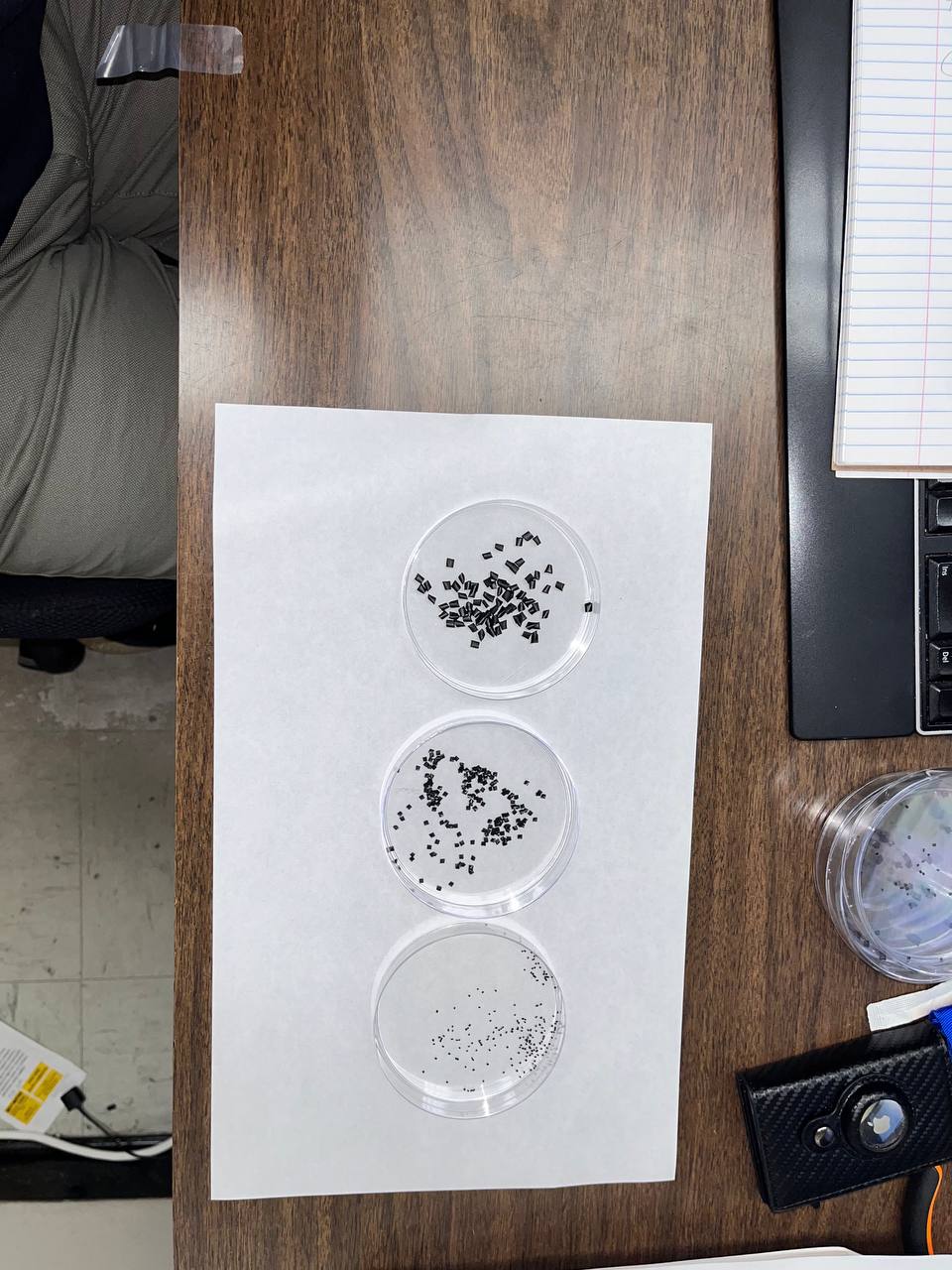


***Fig. SI-11*** *Films for adding into the soil. From the left to right, the films are sized 0.5 - 1 mm, 1 - 3 mm and 3 - 5 mm.*


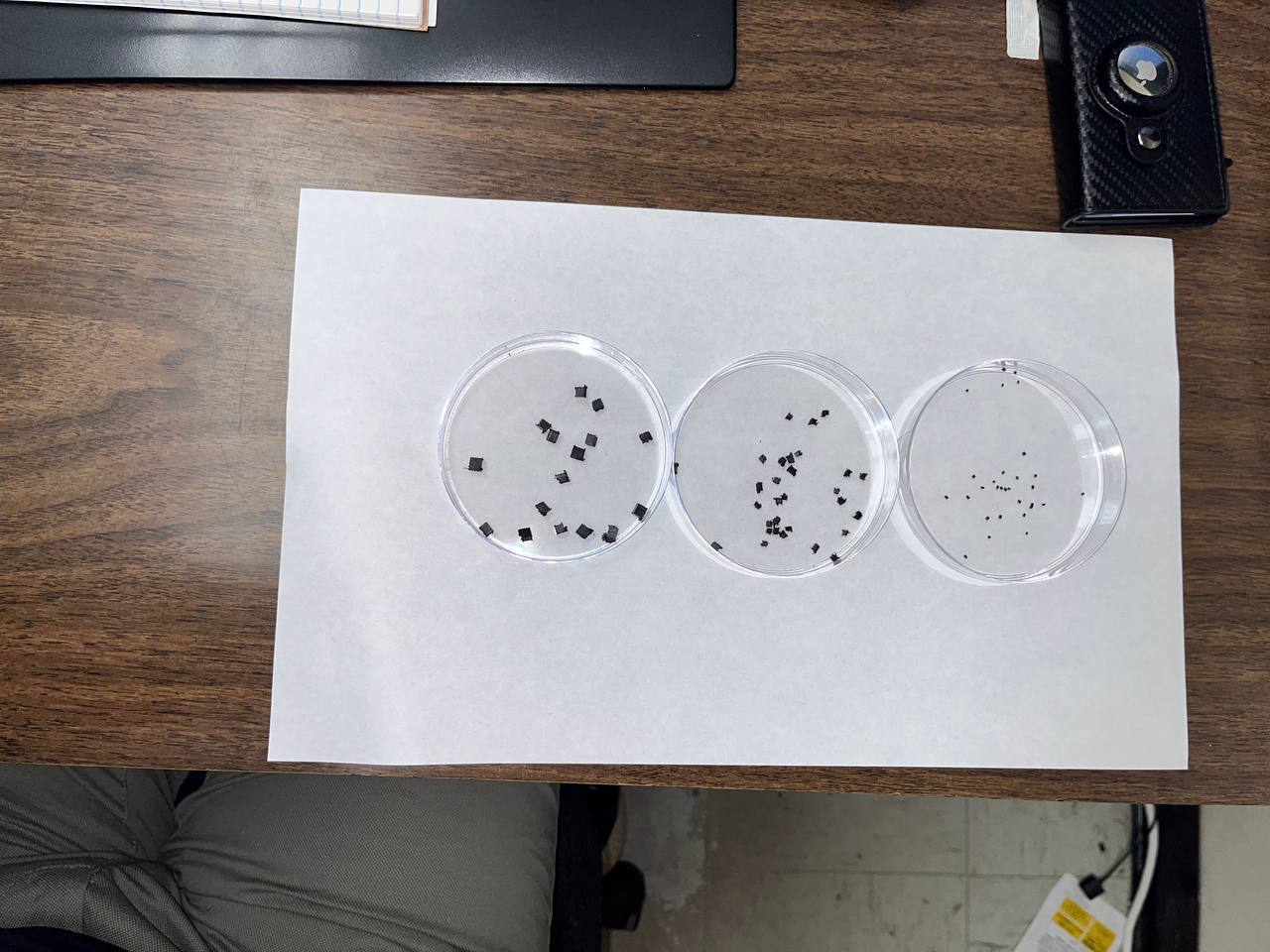


***Fig. SI-12*** *Fragments for adding into the soil. From left to right, the fragments are sized 0.5 - 1 mm, 1 - 3 mm, and 3 - 5 mm.*
